# Older Adult Employment Status and Well-Being: A Longitudinal Bidirectional Analysis

**DOI:** 10.3390/ijerph182312533

**Published:** 2021-11-28

**Authors:** Jonathan L. Chia, Andree Hartanto

**Affiliations:** School of Social Sciences, Singapore Management University, Singapore 178903, Singapore; andreeh@smu.edu.sg

**Keywords:** older adults, employment, well-being, longitudinal analysis

## Abstract

Mixed findings in the literature on the effects of older adult employment on well-being and the reciprocal influence of well-being on employment suggest the need for more careful methodology in teasing out this relationship. Moreover, as previous research has shown that different domains of well-being relate to constructs differently, more nuanced definitions of well-being may be appropriate. The present study examined the longitudinal bidirectional associations of employment and different domains of well-being, controlling for stable within-person variables. The present study sampled older adults from the Midlife Development in the US study at three timepoints on employment status and well-being, specifically psychological, social, and subjective well-being. A Random-Intercept Cross-lagged Panel Model (RI-CLPM) approach was employed to determine the longitudinal bidirectional influence of employment and domains of well-being. Results showed that employment status was not associated with various well-being domains at a later time point. Results also showed that greater well-being, specifically in meaningfulness of society and personal growth, was associated with being employed at a later time point.

## 1. Introduction

The proportion of older persons aged 55 and above is increasing at an unprecedented rate. This observed trend may be in part explained by improvements in the current standard of living and modern medicine [1,2]. In fact, life expectancy has increased by more than 6 years within the span of the last two decades (2000–2019; [3]). Despite an increase in life expectancy, this does not equate to a corresponding increase in quality of life. Indeed, an increase in life expectancy has been associated with increased risk of diseases such as cardiovascular diseases, osteoporosis, hyperlipidemia, cancer, disability, and dementia [4,5,6]. Preventive and nonpharmacological approaches to tackle these issues and improve the quality of life among older adults have consequently garnered significant attention in recent years [7,8,9]. The concept of active ageing embodies the spirit of these nonpharmacological approaches.

The basic tenets that underpin the concept of active ageing have been longstanding in gerontological theories. One such example is the Activity Theory of Ageing [10,11]. In the seminal work by Havighurst and colleagues [10,11], the Activity Theory of Ageing examines the experience of an elderly person when he/she encounters biopsychosocial decline in one’s later years (e.g., retirement, role loss, and death). Cavan and colleagues [10] posited that reduction in well-being was largely due to maladaptive adjustments to said decline. In other words, in a world where activity-oriented, work-related lifestyle serves as a standard of well-being, an important issue of life in one’s later years is the dissociation caused when one’s present state of living no longer conforms to this standard [12]. Hence, a solution to this problem is to adjust one’s life to reflect this standard again, taking on a number and variety of “productive” roles. The theory asserts a positive relationship between older adult’s participation in social activities and life satisfaction, stemming from the underlying notion that beyond from physical decline, psychosocial needs remain [13]. Consequently, older adults would thrive in group and community affairs, which are unfortunately often blocked by social norms such as forced retirement [14].

### 1.1. Older Adult Employment and Well-Being

Older adult employment has been posited to carry a host of benefits, beyond productivity, and economic gain [15,16]. Using a sample of close to 8000 mid-to-older adults, growth curve models assessing the effects of productive activities on mental health trajectories revealed protective effects [17]. Specifically, full-time employment and low-level volunteering had an independent protective effect against decline in psychological well-being, with the joint participation of both resulting an in even stronger protective effect [17]. Similarly, research on older adult employment and well-being have suggested that working beyond traditional retirement ages may yield mental health benefits. In a systematic literature review by Maimaris and Lock [18], five of the seven cross-sectional studies examined reported improved mental health among individuals who had engaged in work beyond retirement age; the other two studies reported non-significant benefits. Of the three longitudinal studies reviewed, all had reported significant benefits of work on mental health [18]. Despite these promising findings, more recent literature has been mixed. In a recent systematic literature review by Baxter and colleagues [19] examining the effects of employment beyond traditional working age on mental health, of the five studies examined: one had reported positive effects, three a neutral effect, and two an adverse effect. These mixed findings may be a result of confounding variables such as individual lifestyle, socioeconomic status, and personality factors [18], with different studies having controlled for a different set of confounds. Further, individual domains of well-being may be associated with employment differently [20,21]. Examining the effects of employment on well-being as a singular construct may fail to capture a potentially more nuanced relationship.

Research has also suggested a reserve direction in the employment–well-being relationship; that is, well-being influences employment. The reverse causal or selection hypothesis presupposes that unemployment may be a consequence of poor mental health and well-being. In a meta-analysis involving 49 independent samples, poor mental health was shown to have a significant negative effect on re-employment [22]. Similarly, poor well-being has been associated with poorer job performance and increased likelihood of future unemployment [23,24]. Indeed, poorer well-being may lead to unsatisfactory job behaviors such as absenteeism, leading to dismissal [25]. Contrary to these findings, however, several studies have suggested the opposite effect of poorer well-being predicting employment [26,27]. Results indicated that a greater loss in well-being motivated efforts towards re-employment so as to reinstate one’s sense of well-being [26]. In line with this observation, when the unemployment rate among an individual’s reference group was higher, there was a smaller drop in well-being among the unemployed, and a reduction in job search intensity [27]. That is, for the unemployed, when relevant others were also jobless, there was a smaller drop in well-being and a reduced intensity in job search, in comparison to when relevant others were employed.

The current body of literature examining the effects of older adult employment on well-being has been mixed. Literature on the reverse effect of well-being on employment have also been mixed. In order to better understand the relationship between older adult employment and well-being, and considering potential policy and intervention implications, this necessitates further examination.

### 1.2. The Current Study

Three key challenges in the current body of literature have prompted this study. First, the mixed findings on the effects of older adult employment on well-being suggests the need for more careful methodology in teasing out this relationship. Well-being is a result of a host of factors, such as lifestyle, personality, and socioeconomic status. Accurately examining the impact of employment on well-being poses a challenge, given the number of potential factors involved and the individual differences in these factors. While studies have attempted to control for socioeconomic variables, demographic factors, and pre-existing medical conditions [28,29,30,31,32], no gerontology study to our knowledge had controlled for multiple trait-like constructs such as personality, temperament, childhood socioeconomic status, etc. These variables may very well confound said relationship, contributing to the mixed results in the literature. Second, among studies that explored the reverse direction of well-being on employment, these findings have also been mixed. Once again, these studies had not controlled for multiple trait-like constructs. These variables may likewise confound the effects of well-being on employment. Thus, a robust methodology using more stringent controls for potential confounds of this reverse relationship is warranted. Third, as the subdomains of well-being may relate to employment differently, exploring the association between employment and well-being as a single construct may fail to capture these nuances. Hence, in order to understand the relationship between older adult employment and well-being in its entirety, investigating how employment may be related to different subdomains of well-being is imperative.

## 2. Materials and Methods

### 2.1. Participants

The current study comprises of older adults from the first [33], second [34], and third [35] wave of the Midlife in the United States (MIDUS) survey study. The MIDUS study was conducted on a random-digit-dial sample of non-institutionalized, English-speaking adults. The survey portion of MIDUS 1 (Timepoint 1; T1), MIDUS 2 (Timepoint 2; T2), and MIDUS 3 (Timepoint 3; T3) were conducted between 1995 and 1997; 2004 and 2006; and 2013 and 2015, respectively. In accordance with studies on older adults, individuals below the age of 55 were excluded from the study [36,37]. Individuals who were reported to be full-time students were also excluded from the study. The resultant sample consisted of 695 individuals. A sample size of more than 200 participants has been suggested to be necessary for complex structural equation modelling [38], of which RI-CLPM is a specification of. Given that more complex models such as the model examined in the current study would require a larger sample, the current sample size of 695 participants was judged to be appropriate, similar to past research employing a three-wave RI-CLPM approach [39,40,41]. Table 1 summarizes the descriptive statistics of our sample. Data and materials of the current study may be accessed via the Inter-University Consortium for Political and Social Research website.

### 2.2. Measures

***Employment status***. Employment status was recorded at three timepoints—MIDUS 1, MIDUS 2, and MIDUS 3—and was coded as unemployed (0) or employed (1).

***Social well-being***. Social well- being was assessed using a 15-item inventory [42], at three timepoints. The 15-item scale measures social well-being through five domains: social coherence (e.g., “I cannot make sense of what’s going on in the world”), social integration (e.g., “I don’t feel I belong to anything I’d call a community”), social acceptance (e.g., “People do not care about other people’s problems”), social contribution (e.g., “I have something valuable to give to the world”), and social actualization (e.g., “Society has stopped making progress”. Response was captured by a 7-point Likert scale (1 = Strongly agree to 7 = Strongly disagree). Higher scores indicated greater social well-being.

***Psychological well-being***. Psychological wellbeing was assessed using an 18-item scale [43], at three timepoints. The scale captures psychological well-being across six domains: positive relations with others (e.g., “Maintaining close relationships has been difficult and frustrating for me”), self-acceptance (e.g., “I like most parts of my personality”), autonomy (e.g., “I have confidence in my own opinions, even if they are different from the way most other people think”), personal growth (e.g., “For me, life has been a continuous process of learning, changing, and growth”), environmental mastery (e.g., “The demands of everyday life often get me down”), and purpose in life (e.g., “Some people wander aimlessly through life, but I am not one of them”). Response was captured by a 7-point Likert scale (1 = Strongly agree to 7 = Strongly disagree). Higher scores indicated greater psychological well-being.

***Subjective well-being***. Subjective well-being was assessed through positive and negative affect. Positive affect was assessed using a 6-item inventory [44], at three timepoints. The six items measure frequency of experienced positive affect over the past 30 days (e.g., “During the past 30 days, how much of the time did you feel cheerful?”). Response was captured on a 5-point Likert scale (1 = All of the time to 5 None of the time). Lower scores indicated greater positive affect. Negative affect was assessed using a 6-item inventory [44], at three timepoints. The six items measure frequency of experienced negative affect over the past 30 days (e.g., “During the past 30 days, how much of the time did you feel so sad nothing could cheer you up?”). Response was captured on a 5-point Likert scale (1 = All of the time to 5 None of the time). Lower scores indicated greater negative affect.

***Covariates***. Covariates included in this study were sex, age, education, ethnicity, and health. Sex and ethnicity were coded as a dichotomous variable (male vs. female; white vs. non-white). Education was rated on a scale of 1 (No school) to 12 (Ph.D, ED. D, MD, LLB, LLD, JD, or other professional degree). Health was assessed using a self-reported measure on physical health (i.e., “In general, would you say your physical health is...”), with response being capture on a 5-point Likert scale (1= poor to 5 = Excellent). Demographic covariates used were assessed at baseline. Health was assessed at each of the three timepoints.

### 2.3. Statistical Analysis

**Figure 1:**
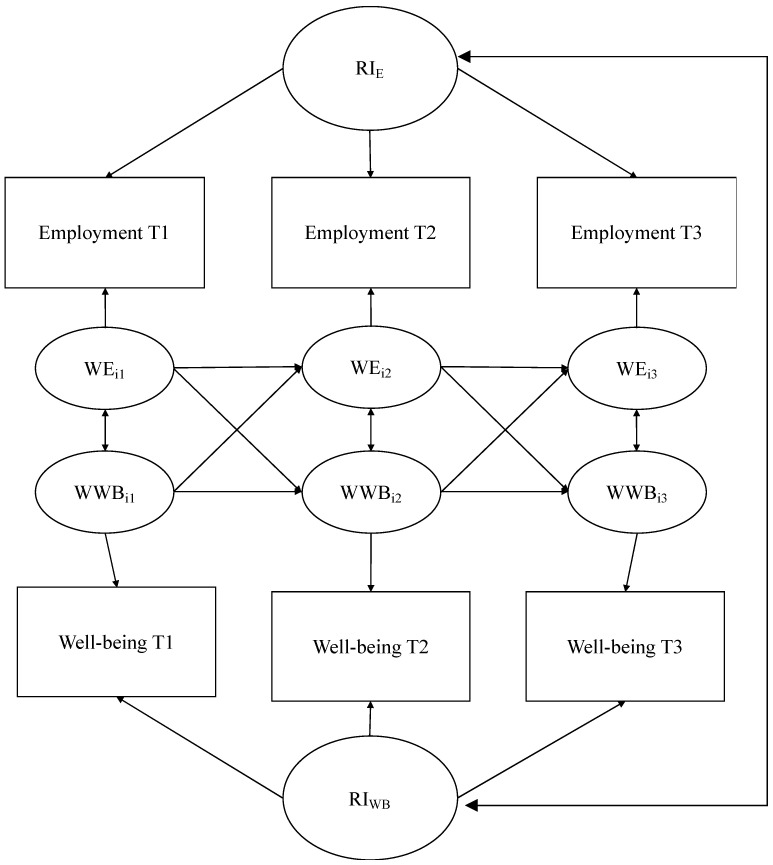
Graphic representations of the random intercept cross-lagged panel model (RI-CLPM) examined in the study. Squares represent observed variables (i.e., employment status, well-being scores), circles denote latent variables. WEit denotes within-person employment status and WWBit denotes within-person well-being domain, of unit i at timepoint t. RI_E_ denotes the random intercept of employment and RI_WB_ denotes the random intercept of well-being.

The present study aimed to examine longitudinal bidirectional associations between employment and well-being—specifically psychological, social, and subjective well-being—controlling for within-person trait-like constructs. In order to achieve this, we employed a Random-Intercept Cross-Lagged Panel Model (RI-CLPM) approach in our analyses. The standard Cross-Lagged Panel Model (CLPM) is a popular method used to analyze interactions and bidirectional associations between longitudinally assessed variables, estimating autoregressive and cross-lagged effects within and between variables of interest, respectively [45]. RI-CLPM is an alternative specification to the CLPM, accounting for within-person autoregressive and cross-lagged effects [45,46]. We conducted our analyses in R version 3.6.3 [47] using three-wave random-intercept cross-lagged panel model (RI-CLPM). Correlations were computed using *Hmisc* version 4.5-0 [48] and RI-CLPM was computed using *lavaan* version 0.6-7 [49]. The RI-CLPM included both autoregressive and cross-lagged paths between employment and well-being, as well as reciprocal paths between well-being and employment. Each domain of well-being and its relation to employment was separately examined. First, associations between employment and five domains of social well-being were examined: meaningfulness of society, social integration, social actualization, social contribution, and acceptance of others. Next, associations between employment and the six domains of psychological well-being were examined: positive relations with others, self-acceptance, autonomy, personal growth, environmental mastery, and purpose in life. Last, associations between employment and subjective well-being were examined: positive affect, negative affect. The RI-CLPM used in the current investigation can be found in Figure 1.

We ran the unconstrained RI-CLPM controlling for time-invariant covariates (TICs) and time-varying covariates (TVCs). Although autoregressive and cross-lagged paths may be constrained to be equal across time, this is not a general precondition for the model to be applied; changes in the magnitude or either autoregressive or cross-lagged path suggests shifts in the developmental system [45]. Lifespan theories suggest changing psychosocial developmental needs from age 55 and beyond [50,51,52]. Hence, in line with existing theories, it was assumed that the reciprocal influence between employment and well-being differed between timepoints, and cross-lagged paths were not constrained. Similarly, owing to the social convention of forced retirement beyond a certain age [14,53,54], and rapid changes relating to physical deterioration may result in sudden changes in well-being [55,56,57], we did not constrain autoregressive paths. TICs specified in the model were sex, age, education, and ethnicity; the TVC specified in the model was health. Although statistical methods like the RI-CLPM control for the influence of any implicit/unobserved third variable that does not change across time (e.g., personality, demographics, lifestyle, etc.), some TICs, while still maintaining their value, may assert a different influence across time. For example, sex, while generally remaining unchanged, may exert a different influence on employment and well-being across time [58,59]. Consequently, by introducing TICs such as sex, age, education, and ethnicity as observed third variables in our models, we avoid assuming that these TICs have a uniformed influence on variables of interest across time [60]. As opposed to TICs, TVCs change across time and have different values at each timepoint. As health was expected to differ across lifespan, it was introduced into our models as a TVC, reflected by separate health measures at each timepoint. To test the various model, we adopted the most frequently reported model indices of Comparative Fit Index (CFI) and root-mean-square error of approximation (RMSEA), with criterion values of around 0.95 and up to 0.08, respectively [61].

## 3. Results

Means, standard deviations, and zero-order correlations for social well-being, psychological well-being, and subjective well-being may be found in Appendix A (Table A1, Table A2 and Table A3). Zero-order correlations revealed small to medium correlations between the various domains of social well-being and across timepoints (*r* = 0.06 to 0.58). A similar pattern was observed among psychological (*r* = 0.05 to 0.62) and subjective well-being domains (*r* = 0.34 to 0.64).

### 3.1. Employment and Social Well-Being

***Meaningfulness of Society (Social Coherence).*** The RI-CLPM with employment and meaningfulness of society was found to be of good fit (χ^2^(31) = 80.681, *p* < 0.05; CFI = 0.963, TLI = 0.906, RMSEA = 0.048, 90% CI (0.035, 0.061)). Results indicate a significant autoregressive influence of employment between timepoint 1 and 2 (*β*_T1T2_ = 0.32, *p* < 0.001) and timepoint 2 and 3 (*β*_T2T3_ = 0.28, *p* <0.001). Results also indicated a significant cross-lagged influence. Meaningfulness of society at timepoint 2 was found to predict employment at timepoint 3 (*β*_T2T3_ = 0.03, *p* = 0.015). No other paths were significant.

***Social integration.*** The RI-CLPM with employment and social integration was found to be of good fit (χ^2^(31) = 81.536, *p* < 0.05; CFI = 0.961, TLI = 0.902, RMSEA = 0.048, 90% CI (0.036, 0.061)). Results indicate a significant autoregressive influence of employment across timepoints (*β*_T1T2_ = 0.33, *β*_T2T3_ = 0.29, *p* < 0.001). Results also indicated a significant autoregressive influence of social integration across timepoints (*β*_T1T2_ = 0.18, *p* = 0.015; *β*_T2T3_ = 0.19, *p* = 0.044). No other paths were significant.

***Social actualization.*** The RI-CLPM with employment and social actualization was found to be of good fit (χ^2^(31) = 81.173, *p* < 0.05; CFI = 0.955, TLI = 0.886, RMSEA = 0.048, 90% CI (0.036, 0.061)). Results indicate a significant autoregressive influence of employment across timepoints (*β*_T1T2_ = 0.34, *β*_T2T3_ = 0.29, *p* < 0.001). Results also indicated a significant autoregressive influence of social actualization between timepoint 1 and 2 (*β*_T1T2_ = 0.25, *p* < 0.001). No other paths were significant.

***Social contribution.*** The RI-CLPM with employment and social contribution was found to be of good fit (χ^2^(31) = 82.324, *p* < 0.05; CFI = 0.962, TLI = 0.905, RMSEA = 0.049, 90% CI (0.036, 0.062)). Results indicate a significant autoregressive influence of employment across timepoints (*β*_T1T2_ = 0.33, *β*_T2T3_ = 0.28, *p* < 0.001). Results also indicated a significant autoregressive influence of social contribution across timepoints (*β*_T1T2_ = 0.25, *β*_T2T3_ = 0.25, *p* < 0.01). No other paths were significant. 

***Acceptance of Others (Social Acceptance).*** The RI-CLPM with employment and acceptance of others was found to be of good fit (χ^2^(31) = 78.216, *p* < 0.05; CFI = 0.957, TLI = 0.891, RMSEA = 0.047, 90% CI (0.034, 0.060)). Results indicate a significant autoregressive influence of employment across timepoints (*β*_T1T2_ = 0.33, *β*_T2T3_ = 0.29, *p* < 0.001). Results also indicated an autoregressive influence of acceptance of others between timepoint 1 and 2 (*β*_T1T2_ = 0.19, *p* < 0.01). No other paths were significant.

Results from RI-CLPM analyses of employment and social well-being maybe found in Appendix B (Table A4).

### 3.2. Employment and Psychological Well-Being

***Positive relations with others.*** The RI-CLPM with employment and positive relations with others was found to be of good fit. (χ^2^(31) = 92.540, *p* < 0.05; CFI = 0.956, TLI = 0.889, RMSEA =0.053, 90% CI (0.041, 0.066)). Results indicate a significant autoregressive influence of employment (*β*_T1T2_ = 0.33, *β*_T2T3_ = 0.29, *p <* 0.001). No other paths were significant. 

***Self-acceptance.*** The RI-CLPM with employment and self-acceptance was found to be of good fit. (χ^2^(31) = 97.316, *p* < 0.05; CFI = 0.950, TLI = 0.875, RMSEA = 0.055, 90% CI (0.043, 0.068)). Results indicate a significant autoregressive influence of employment (*β*_T1T2_ = 0.32, *β*_T2T3_ = 0.29, *p <* 0.001). Results also indicated a significant autoregressive influence of self-acceptance between timepoint 2 and 3 (*β*_T2T3_ = 0.21, *p <* 0.01). No other paths were significant.

***Autonomy.*** The RI-CLPM with employment and autonomy was found to be of good fit. (χ^2^(31) = 75.044, *p* < 0.05; CFI = 0.959, TLI = 0.897, RMSEA = 0.045, 90% CI (0.032, 0.058)). Results indicate a significant autoregressive influence of employment (*β*_T1T2_ = 0.33, *β*_T2T3_ = 0.28, *p* < 0.001). No other paths were significant.

***Personal growth.*** The RI-CLPM with employment and personal growth was found to be of good fit. (χ^2^(31) = 86.057, *p* < 0.05; CFI = 0.956, TLI = 0.890, RMSEA = 0.051, 90% CI (0.038, 0.063)). Results indicate a significant autoregressive influence of employment (*β*_T1T2_ = 0.32, *β*_T2T3_ = 0.28, *p <* 0.001) across timepoints, and a significant autoregressive influence of personal growth between timepoint 2 and 3 (*β*_T2T3_ = 0.20, *p <* 0.01). Results also indicated a significant cross-lagged influence. Personal growth at timepoint 1 was found to predict employment at timepoint 2 (*β*_T1T2_ = 0.04, *p <* 0.01), and personal growth at timepoint 2 was found to predict employment at timepoint 3 (*β*_T2T3_ = 0.02, *p* = 0.035). No other paths were significant.

***Environmental mastery.*** The RI-CLPM with employment and environmental mastery was found to be of acceptable fit. (χ^2^(31) = 95.268, *p* < 0.05; CFI = 0.944, TLI = 0.860, RMSEA = 0.055, 90% CI (0.042, 0.067)). Results indicate a significant autoregressive influence of employment (*β*_T1T2_ = 0.33, *β*_T2T3_ = 0.29, *p <* 0.001). Result also indicate a significant autoregressive influence of environmental master from timepoint 1 to timepoint 2 (*β*_T1T2_ =0.17, *p* =0.009). No other paths were significant.

***Purpose in life.*** The RI-CLPM with employment and purpose in life was found to be of good fit. (χ^2^(31) = 79.635, *p* < 0.05; CFI = 0.955, TLI = 0.888, RMSEA = 0.048, 90% CI (0.035, 0.061)). Results indicate a significant autoregressive influence of employment (*β*_T1T2_ = 0.33, *β*_T2T3_ = 0.29, *p <* 0.001). Results also indicate a significant autoregressive influence of purpose in life from timepoint 2 to timepoint 3 (*β*_T2T3_ = 0.21, *p* = 0.002). No other paths were significant.

Results from RI-CLPM analyses of employment and psychological well-being are presented in Appendix C (Table A5).

### 3.3. Employment and Subjective Well-Being

***Positive Affect.*** The RI-CLPM with employment and positive affect was found to be of acceptable fit. (χ^2^(31) = 106.079, *p* < 0.05; CFI = 0.945, TLI = 0.861, RMSEA = 0.059, 90% CI (0.047, 0.072)). Results indicate a significant autoregressive influence of employment (*β*_T1T2_ = 0.33, *β*_T2T3_ = 0.30, *p* < 0.001). Results also indicated a significant autoregressive influence of positive affect between timepoint 2 and 3 (*β*_T2T3_ = 0.43, *p* < 0.001). No other paths were significant.

***Negative Affect.*** The RI-CLPM with employment and negative affect was found to be of adequate fit. (χ^2^(31) = 109.253, *p* < 0.05; CFI = 0.938, TLI = 0.844, RMSEA = 0.060, 90% CI (0.048, 0.072)). Results indicate a significant autoregressive influence of employment (*β*_T1T2_ = 0.33, *β*_T2T3_ = 0.29, *p <* 0.001). No other paths were significant.

Results from RI-CLPM analyses of employment and subjective well-being are presented in Appendix D (Table A6).

## 4. Discussion

The present study sought to shed light on the inconsistent findings on employment and well-being in the literature. First, given how various within-person factors may influence associations between the variables of interest, the present study attempted to control for within-person trait-like constructs by employing a RI-CLPM approach in our analyses. The standard CLPM has been subject to criticism in recent years, prompting the improved extension to the method that is the RI-CLPM. The central issue with the CLPM is its failure to examine within-person effects; it assumes individuals vary around a common group mean in the examined variable over time [45,46,62,63]. The RI-CLPM has hence been presented as an alternative specification to the CLPM, addressing this concern by taking into account within-person autoregressive and cross-lagged effects, allowing each individual to vary around their individual, stable, trait-like levels over time (see [45,46]). Second, as domains of well-being may relate to employment differently, the present study explored the associations between employment and domains of psychological, social, and subjective well-being, rather than well-being as a single construct. Our findings are discussed below in three sections.

### 4.1. Preliminary Analyses and Autoregressive Influence

Preliminary correlational analyses revealed weak to moderate correlation between domains of well-being. This supports the notion of well-being domain uniqueness, and how the different domains of well-being may relate to constructs differently [20,21]. Regarding autoregressive influences, a lack of uniform influence between the various well-being domains across time was observed. This suggests that different domains of well-being develop differently across older adulthood, in line with previous research on well-being developmental changes [55,56,57]. Contrary to well-being domains, autoregressive influence was observed consistently for employment across timepoints. This suggests that employment in the past is likely to predict employment in the future. While this appears to be in opposition to the social convention of forced retirement [14,53,54], it may not necessarily be the case. The normative retirement age may vary between occupations and job characteristics. For example, level of physically strain imposed by the occupation [64], autonomy in job task [64], and organization structure [65,66,67] have been found to influence retirement age. Forced retirement may also relate to full-time employment rather than part-time employment. Indeed, government policies have sort to support older adult part-time employment [68]. The variability in retirement age, together with support for part-time employment among older adults, may have resulted in the autoregressive influence of employment observed. 

### 4.2. Significant Cross-Lagged Influence

Bidirectional cross-lagged findings from our study suggest greater social and psychological well-being to predict older adult employment, specifically, meaningfulness of society and personal growth respectively. These significant cross-lagged influences seem to lend support to previous studies positing well-being to predict employment [22,23,24,25], at least among older adults.

Meaningfulness of society is the perception that life is generally sensible, understandable, and controllable [42]. Individuals high on meaningfulness of society are concerned about the kind of world they live in, and maintain the desire to make sense of life [42]. Hence, older adults who struggle to understand or adapt to current society may feel disheartened and experience reduced levels of well-being in this domain [67,69,70]. Consequently, they may choose to withdraw socially and in terms of employment [65,66,67]. Of note, however, this influence was only observed between timepoint 2 and timepoint 3. Observation of the descriptive statistics suggest that a steeper decline in meaningfulness of society in later life, between timepoint 2 and timepoint 3 (compared to the decline between timepoint 1 and 2), might have allowed for this relationship to become more pronounced.

Personal growth relates to well-being from the sense that one is able to continually develop, grow and expand as a person [43]. Results from this study suggest personal growth to predict future employment. Indeed, personal growth has been found to predict feelings to interest and commitment [71]. This notion has also been supported by literature on other similar psychological constructs such as personal expressiveness [72] and flow [73]. As such, older adults who have experienced greater personal growth may express more interest in activities, extending to gainful employment.

### 4.3. Non-Significant Cross-Lagged Influence

Bidirectional cross-lagged results from our study suggest older adult employment to have a neutral influence on domains of well-being. That is, when controlling for within-person cross-time stabilities and correcting for potentially inflated observations, older adult employment did not significantly influence well-being in either direction. These findings are in line with a recent growing body of literature supporting a neutral influence of older adult employment on well-being, across various well-being indicators such as depression [30], current and life-as-a-whole life satisfaction [28], quality of life [30], and affect relating to common mental disorders [32]. Our findings suggest that previously observed effects of employment on well-being may be a result of the influence of individual differences in within-person stable factors. An equally plausible explanation could be differences in sample characteristics—with the current study focusing exclusively on employment among older adults—and the way well-being had been previously operationalized.

Null findings were also observed from investigations on the reciprocal relationship of other well-being domains (i.e., social integration, social actualization, social contribution, acceptance of others, positive relations with others, self-acceptance, autonomy, environmental mastery, purpose in life, positive affect, and negative affect), apart from meaningfulness of society and personal growth, on older adult employment. That is, when controlling for within-person cross-time stabilities, these domains of well-being did not significantly influence older adult employment in either direction. Our findings suggest that previously observed influence of poorer well-being predicting subsequent employment may be a result of the influence of within-person stable factors, such as locus of control [74] and temperament [75]. Again, an equally plausible explanation could be differences in sample characteristics and the way well-being had been previously operationalized.

In summary, the present study’s findings shed new light on the discourse surrounding the relative influence of older adult employment on well-being and the reciprocal relationship. On face value, the idea of older adult employment seems promising; in a world where activity-oriented and work-related lifestyle serves as a standard of well-being, older adult employment may be a potentially mechanism to improved well-being among seniors.

However, recent literature reporting null findings across various well-being indicators, together with this study’s null findings across various domains of well-being, suggest otherwise. The health and well-being trajectory of older adults vary greatly between persons. As such, in gerontology research, many within-person factors have been studied as potential predictors of successful ageing, such as personality, attitudes, dispositions, and lifestyles [76,77,78]. When taking these factors into account, results from this study suggest any influence of employment on well-being negated. While this negated influence appears to extend to the reciprocal influence of well-being on employment, there were two notable exceptions: meaningfulness of society and personal growth was found to predict employment. Previous literature suggest that a reduced meaningfulness of society may indicate one’s struggle to adapt to changes to the environment, which may lead to greater social withdrawal, extending to employment. Previous literature also suggests that a reduced personal growth may lead to a reduction in activity interest, which may similarly lead to greater withdrawal socially and in terms of employment.

## 5. Study Strengths and Limitations

To our best knowledge, the present study is one of the first studies to explore the longitudinal association of employment and well-being among older adults. Because we used a RI-CLPM, we were able to study associations in a bi-directional manner, while controlling for stable within-person factors. This study also explored a more nuanced relationship between employment and the different well-being domains.

The study has several limitations. First, this study does not distinguish between the type of occupation held. It may be possible for the reported findings to different across different types of occupations. For example, older adults working in jobs that help the less fortunate may report greater sense of well-being at a later timepoint, an observation similar to the effects of volunteerism [79]. In the same vein, it may be possible that increased meaningfulness of society and personal growth lead to employment in specific types of occupation. Second, this study does not distinguish between job characteristics. It may be possible that different job characteristics, even within the same occupation, may exert different influences on well-being, and vice versa. For example, occupations with exposure to trauma (e.g., bullying at work, or handling corpses) may result in poorer well-being at a later timepoint [80]. Third, although this study looked at employment in relation to various well-being domains, these domains are by no means exhaustive. For example, another domain of well-being not considered in this study is spirituality and religiosity well-being [81]. It may be possible that employment exerts a certain influence over other domains of well-being, and vice versa. An interaction between type of occupation, job characteristics, and other domains of well-being may also be possible. Forth, the present study examined employment and well-being among predominantly white older adults living in the United States. This relationship may differ cross-culturally [82,83].

## 6. Conclusions

As stressed by several researchers (e.g., [17,19]), knowledge about the temporal relationship between employment and well-being has important implications. Understanding this relationship can inform policymakers looking to introduce programs and legislation aimed at facilitating better mental health outcomes and quality of life among older adults. Our findings suggests that when within-person factors are accounted for, employment did not result in significantly greater or lesser well-being. Introducing older adult employment as an intervention to improve well-being among seniors may not be an effective strategy. While it may be that conventional employment arrangements are not structured to elicit the benefits illustrated by previous theories (e.g., [10]), and that a modified employment structure may yield theorized benefits, these questions are beyond the current research. Our findings, however, do suggest that well-being, specifically meaningfulness of society and personal growth, leads to employment among older adults. Nevertheless, caution should be taken in assuming the universality of these influence across occupation types, job characteristics, and culture. This research highlights the importance of controlling for within-person stable variables when examining temporal influence of predictors of interest on ageing outcomes. Future research examining the ageing process may wish to consider variables of interest within within-person contexts.

## Figures and Tables

**Table 1:** Descriptive statistics of study sample.

		*M* (SD) *or %*	Range
Demographic information			
	Mean Age (years)	60.90 (4.78)	55–74
	Sex (% female)	56%	
	Race (% White)	95%	
	Marital status (% married)	73%	
	Education	7.37 (2.43)	1–12
Health			
	Health at Timepoint 1	3.62 (0.88)	1–5
	Health at Timepoint 2	3.60 (0.90)	1–5
	Health at Timepoint 3	3.25 (1.03)	1–5
Timepoint 1			
	Employed	70.5%	
	Unemployed	29.5%	
Timepoint 2			
	Employed	47.5%	
	Unemployed	52.5%	
Timepoint 3			
	Employed	23.6%	
	Unemployed	76.4%	

*Note*. *n* = 695. Mean (M) and standard deviation (SD); SDs are shown in parentheses. Education attainment was rated on a scale of 1 (No school) to 12 (Ph.D, ED. D, MD, LLB, LLD, JD, or other professional degree).

## Data Availability

Data and materials of the current study may be accessed via the Inter-University Consortium for Political and Social Research website, https://www.icpsr.umich.edu/web/pages/NACDA/midus.html (accessed on 31 August 2021).

## Appendix A

**Table A1:** Means, Standard Deviation, and Zero-order Correlations among Social Well-Being Domains.

		*M* (SD)	1	2	3	4	5	6	7	8	9	10	11	12	13	14	15
Timepoint 1																	
1	Meaningfulness of society	8.95 (3.2)	-	0.18	0.23	0.28	0.36	0.54	0.23	0.15	0.28	0.31	0.49	0.18	0.16	0.28	0.22
2	Social integration	15.50 (4.24)	0.18	-	0.35	0.43	0.29	**0.06**	0.56	0.25	0.31	0.21	0.13	0.48	0.24	0.23	0.16
3	Acceptance of others	14.17 (3.39)	0.23	0.35	-	0.24	0.47	0.15	0.28	0.46	0.19	0.31	0.14	0.24	0.38	0.14	0.23
4	Social contribution	15.81 (3.74)	0.28	0.43	0.24	-	0.29	0.25	0.36	0.25	0.55	0.26	0.19	0.37	0.19	0.46	0.17
5	Social actualization	12.43 (4.20)	0.36	0.29	0.47	0.29	--	0.22	0.21	0.29	0.17	0.47	0.25	0.22	0.20	0.18	0.36
Timepoint 2																	
6	Meaningfulness of society	9.11 (3.11)	0.54	**0.06**	0.25	0.25	0.22	-	0.20	0.21	0.35	0.47	0.51	0.21	0.17	0.33	0.24
7	Social integration	15.75 (3.82)	0.23	0.56	0.28	0.36	0.21	0.20	-	0.35	0.51	0.27	0.19	0.57	0.24	0.38	0.17
8	Acceptance of others	14.85 (3.10)	0.15	0.25	0.46	0.25	0.29	0.21	0.35	-	0.27	0.44	0.16	0.26	0.44	0.16	0.24
9	Social contribution	15.64 (3.74)	0.28	0.31	0.19	0.55	0.17	0.35	0.51	0.27	-	0.27	0.26	0.42	0.16	0.58	0.13
10	Social actualization	12.88 (3.99)	0.31	0.21	0.31	0.26	0.47	0.47	0.27	0.44	0.27	-	0.26	0.22	0.27	0.18	0.40
Timepoint 3																	
11	Meaningfulness of society	8.27 (3.05)	0.49	0.13	0.14	0.19	0.25	0.51	0.19	0.16	0.26	0.26	-	0.26	0.26	0.38	0.44
12	Social integration	15.29 (3.94)	0.18	0.48	0.24	0.37	0.22	0.21	0.57	0.26	0.42	0.22	0.26	-	0.34	0.47	0.25
13	Acceptance of others	14.43 (3.02)	0.16	0.24	0.38	0.19	0.20	0.17	0.24	0.44	0.16	0.27	0.26	0.34	-	0.23	0.37
14	Social contribution	14.59 (3.76)	0.28	0.23	0.14	0.46	0.18	0.33	0.38	0.16	0.58	0.18	0.38	0.47	0.23	-	0.26
15	Social actualization	11.42 (3.91)	0.22	0.16	0.23	0.17	0.36	0.24	0.17	0.24	0.13	0.40	0.44	0.25	0.37	0.26	-

All correlations were significant (*p* < 0.01) except between integration at timepoint 1 and meaningfulness at timepoint 2 (*p* = 0.15), in bold.

**Table A2:** Means, Standard Deviation, and Zero-order Correlations among Psychological Well-Being Domains.

		*M* (SD)	1	2	3	4	5	6	7	8	9	10	11	12	13	14	15	16	17	18
Timepoint 1																				
1	Positive Relations with Others	17.00 (3.88)	-	0.39	0.17	0.41	0.36	0.31	0.62	0.37	0.22	0.32	0.30	0.25	0.52	0.32	**0.07**	0.27	0.17	0.23
2	Self-acceptance	17.07 (3.18)	0.39	-	0.23	0.29	0.55	0.23	0.30	0.54	0.15	0.24	0.30	0.16	0.31	0.53	0.13	0.24	0.32	0.18
3	Autonomy	16.79 (3.16)	0.17	0.23	-	0.18	0.25	**0.05**	0.09	0.24	0.46	0.18	0.13	**0.06**	0.11	0.26	0.41	0.25	0.17	**0.06**
4	Personal growth	18.03 (2.99)	0.41	0.29	0.18	-	0.27	0.29	0.35	0.33	0.22	0.48	0.19	0.27	0.32	0.31	0.17	0.46	0.20	0.30
5	Environmental mastery	17.05 (3.21)	0.36	0.55	0.25	0.27	--	0.23	0.28	0.37	0.20	0.19	0.46	0.14	0.25	0.37	0.20	0.19	0.37	0.13
6	Purpose in Life	16.75 (3.53)	0.31	0.23	**0.05**	0.29	0.23	-	0.23	0.16	0.11	0.20	0.17	0.40	0.24	0.20	0.15	0.21	0.16	0.36
Timepoint 2																				
7	Positive Relations with Others	17.71 (3.52)	0.62	0.30	0.09	0.35	0.28	0.23	-	0.46	0.22	0.47	0.42	0.33	0.58	0.38	0.08	0.33	0.21	0.25
8	Self-acceptance	16.97 (3.37)	0.37	0.54	0.24	0.33	0.37	0.16	0.46	-	0.32	0.41	0.48	0.24	0.40	0.57	0.18	0.39	0.37	0.21
9	Autonomy	16.98 (2.88)	0.22	0.15	0.46	0.22	0.20	0.11	0.22	0.32	-	0.25	0.38	0.12	0.20	0.30	0.45	0.23	0.21	0.11
10	Personal growth	17.51 (3.03)	0.32	0.24	0.18	0.48	0.19	0.20	0.47	0.41	0.25	-	0.34	0.31	0.34	0.34	0.20	0.54	0.25	0.29
11	Environmental mastery	17.84 (2.83)	0.30	0.30	0.13	0.19	0.46	0.17	0.42	0.48	0.38	0.34	-	0.22	0.33	0.37	0.16	0.28	0.43	0.20
12	Purpose in Life	16.18 (3.45)	0.25	0.16	**0.06**	0.27	0.14	0.40	0.33	0.24	0.12	0.31	0.22	-	0.29	0.24	0.11	0.32	0.23	0.50
Timepoint 3																				
13	Positive Relations with Others	16.99 (3.63)	0.52	0.31	0.11	0.32	0.25	0.24	0.58	0.40	0.20	0.34	0.33	0.29	-	0.51	0.21	0.48	0.35	0.36
14	Self-acceptance	16.60 (3.41)	0.32	0.53	0.26	0.31	0.37	0.20	0.38	0.57	0.30	0.34	0.37	0.24	0.51	-	0.30	0.44	0.48	0.25
15	Autonomy	16.47 (2.93)	**0.07**	0.13	0.41	0.17	0.20	0.15	0.08	0.18	0.45	0.20	0.16	0.11	0.21	0.30	-	0.26	0.34	0.17
16	Personal growth	17.20 (3.06)	0.27	0.24	0.25	0.46	0.19	0.21	0.33	0.39	0.23	0.54	0.28	0.32	0.48	0.44	0.26	-	0.32	0.38
17	Environmental mastery	16.96 (3.15)	0.17	0.32	0.17	0.20	0.37	0.16	0.21	0.37	0.21	0.25	0.43	0.23	0.35	0.48	0.34	0.32	-	0.29
18	Purpose in Life	15.24 (3.43)	0.23	0.18	**0.06**	0.30	0.13	0.36	0.25	0.21	0.11	0.29	0.20	0.50	0.36	0.25	0.17	0.38	0.29	-

All correlations were significant (*p* < 0.01) except between Autonomy and Purpose in Life at timepoint 1 (*p* = 0.16), Autonomy timepoint 1 and Purpose in Life at timepoint 2 (*p* = 0.14), Positive Relations with Others at timepoint 1 and Autonomy at timepoint 3 (*p* = 0.07), Autonomy at timepoint 1and Purpose in Life at timepoint 3 (*p* = 0.12), in bold.

**Table A3:** Means, Standard Deviation, and Zero-order Correlations among Subjective Well-being Domains.

		*M* (SD)	1	2	3	4	5	6
Timepoint 1								
1	Positive Affect	3.54 (0.64)	-	−0.64	0.50	−0.38	0.47	−0.34
2	Negative Affect	1.40 (0.49)	−0.64	-	−0.38	0.48	−0.37	0.46
Timepoint 2								
3	Positive Affect	3.62 (0.61)	0.50	−0.38	-	−0.55	0.61	−0.34
4	Negative Affect	1.36 (0.45)	−0.38	0.48	−0.55	-	−0.40	0.51
Timepoint 3								
5	Positive Affect	3.50 (0.71)	0.47	−0.37	0.61	−0.40	-	−0.55
6	Negative Affect	1.44 (0.54)	−0.34	0.46	−0.34	0.51	−0.55	-

All correlations were significant (*p* < 0.01).

## Appendix B

**Table A4:** Results from Random-Intercept Cross-Lagged Panel Analysis Modelling Employment and Social Well-Being Domains.

		Meaningfulness of Society			Social Integration			Social Actualization			Social Contribution			Acceptance of Others		
		*β*	*p*	*95% CI*	*β*	*p*	*95% CI*	*β*	*p*	*95% CI*	*β*	*p*	*95% CI*	*β*	*p*	*95% CI*
Employment → ScWB																
	T1 Em → T2 ScWB	−0.23	0.475	−0.88; 0.41	-0.44	0.316	−1.29; 0.42	−0.77	0.080	−1.64; 0.09	−0.46	0.271	−1.27; 0.36	−0.24	0.492	−0.935; 0.45
	T2 Em → T3 ScWB	0.47	0.201	−0.25; 1.19	0.42	0.354	−0.46; 1.30	0.09	0.841	−0.83; 1.02	0.01	0.978	−0.78; 0.80	−0.04	0.920	−0.73; 0.66
SWB → Employment																
	T1 ScWB → T2 Em	−0.00	0.862	−0.03; 0.02	0.01	0.620	−0.01; 0.02	−0.01	0.396	−0.02; 0.01	0.01	0.374	−0.01; 0.03	−0.00	0.936	−0.02; 0.02
	T2 ScWB → T3 Em	0.03	0.015	0.01; 0.05	0.01	0.392	−0.01; 0.03	−0.00	0.766	−0.02; 0.01	0.01	0.204	−0.01; 0.03	−0.01	0.519	−0.03; 0.01
Autoregressive Influence																
	T1 Em → T2 Em	0.32	0.000	0.17; 0.47	0.33	0.000	0.19; 0.48	0.34	0.000	0.19; 0.49	0.33	0.000	0.18; 0.47	0.33	0.000	0.19; 0.48
	T2 Em → T3 Em	0.28	0.000	0.16; 0.39	0.29	0.000	0.18; 0.40	0.29	0.000	0.18; 0.41	0.28	0.000	0.16; 0.39	0.29	0.000	0.18; 0.41
	T1 ScWB → T2 ScWB	0.09	0.196	−0.05; 0.22	0.18	0.015	0.04; 0.33	0.25	0.000	0.13; 0.38	0.25	0.002	0.09; 0.41	0.19	0.002	0.07; 0.31
	T2 ScWB → T3 ScWB	0.01	0.914	−0.15; 0.17	0.19	0.044	0.01; 0.38	0.10	0.205	−0.05; 0.25	0.25	0.001	0.10; 0.40	0.12	0.137	−0.04; 0.27

Em = employment, ScWB = social well-being, T1 = timepoint 1, T2 = timepoint 2, T3 = timepoint 3.

## Appendix C

**Table A5:** Results from Random-Intercept Cross-Lagged Panel Analysis Modelling Employment and Psychological Well-Being Domains.

		Positive Relations			Self-Acceptance			Autonomy			Personal Growth			Environmental Mastery			Purpose in Life		
		*β*	*p*	*95% CI*	*β*	*p*	*95% CI*	*β*	*p*	*95% CI*	*β*	*p*	*95% CI*	*β*	*p*	*95% CI*	*β*	*p*	*95% CI*
Employment → PWB																			
	T1 Em → T2 PWB	0.33	0.334	−0.34; 1.00	−0.12	0.752	−0.86; 0.62	0.30	0.346	−0.32; 0.93	0.09	0.798	−0.61; 0.80	0.34	0.324	−0.34; 10.01	0.18	0.681	−0.67; 1.02
	T2 Em → T3 PWB	0.14	0.753	−0.74; 10.03	−0.17	0.638	−0.89; 0.55	0.68	0.080	−0.08; 10.43	0.36	0.269	−0.28; 10.00	0.01	0.981	−0.71; 0.73	−0.06	0.890	−0.83; 0.72
PWB → Employment																			
	T1 PWB → T2 Em	0.01	0.289	−0.01; 0.04	−0.02	0.217	−0.05; 0.01	0.01	0.683	−0.02; 0.03	0.04	0.006	0.01; 0.06	−0.01	0.546	−0.03; 0.01	−0.01	0.505	−0.03, 0.01
	T2 PWB → T3 Em	0.00	0.839	−0.02; 0.03	−0.00	0.913	−0.02; 0.02	0.02	0.204	−0.01; 0.04	0.02	0.035	0.00 *; 0.04	−0.01	0.564	−0.03; 0.02	0.00	0.751	−0.01; 0.02
Autoregressive Influence																			
	T1 Em → T2 Em	0.33	0.000	0.18; 0.48	0.32	0.000	0.17; 0.47	0.33	0.000	0.18; 0.47	0.32	0.000	0.17; 0.46	0.33	0.000	0.18; 0.48	0.33	0.000	0.18; 0.48
	T2 Em → T3 Em	0.29	0.000	0.18; 0.40	0.29	0.000	0.17; 0.40	0.28	0.000	0.16; 0.40	0.28	0.000	0.17; 0.39	0.29	0.000	0.18; 0.40	0.29	0.000	0.18; 0.40
	T1 PWB → T2 PWB	0.18	0.010	0.04; 0.32	0.14	0.164	−0.06; 0.33	0.08	0.202	−0.04; 0.20	0.10	0.258	−0.07; 0.26	0.17	0.009	0.04; 0.30	0.12	0.093	−0.02; 0.26
	T2 PWB → T3 PWB	0.04	0.726	−0.19; 0.28	0.21	0.002	0.08; 0.34	−0.03	0.726	−0.22; 0.15	0.20	0.004	0.06; 0.34	0.14	0.119	−0.04; 0.31	0.21	0.002	0.08; 0.35

Em = employment, PWB = psychological well-being, T1 = timepoint 1, T2 = timepoint 2, T3 = timepoint 3; *- Lower limit for the 95% confidence interval of Personal Growth was 0.001.

## Appendix D

**Table A6:** Results from Random-Intercept Cross-Lagged Panel Analysis Modelling Employment and Subjective Well-being Domains.

		Positive Affect			Negative Affect		
		*β*	*p*	*95% CI*	*β*	*p*	*95% CI*
Employment → SjWB							
	T1 Em → T2 SjWB	−0.01	0.920	−0.17; 0.15	−0.03	0.578	−0.15; 0.08
	T2 Em → T3 SjWB	0.08	0.214	−0.05; 0.22	−0.03	0.649	−0.15; 0.09
SjWB → Employment							
	T1 SjWB → T2 Em	0.05	0.468	−0.08; 0.18	−0.05	0.591	−0.22; 0.12
	T2 SjWB → T3 Em	−0.02	0.727	−0.13; 0.09	0.02	0.818	−0.14; 0.17
Autoregressive Influence							
	T1 Em → T2 Em	0.33	0.000	0.19; 0.48	0.33	0.000	0.18; 0.48
	T2 Em → T3 Em	0.30	0.000	0.18; 0.41	0.29	0.000	0.18; 0.41
	T1 SjWB → T2 SjWB	0.10	0.348	−0.11; 0.31	0.04	0.624	−0.13; 0.21
	T2 SjWB → T3 SjWB	0.43	0.000	0.26; 0.59	0.17	0.093	−0.03; 0.36

Em = employment, SjWB = subjective well-being, T1 = timepoint 1, T2 = timepoint 2, T3 = timepoint 3.
